# Some Environmental Contaminants Influence Motor and Feeding Behaviors in the Ornate Wrasse (*Thalassoma pavo*) via Distinct Cerebral Histamine Receptor Subtypes

**DOI:** 10.1289/ehp.7983

**Published:** 2005-07-14

**Authors:** Giuseppina Giusi, Rosa Maria Facciolo, Raffaella Alò, Antonio Carelli, Maria Madeo, Pietro Brandmayr, Marcello Canonaco

**Affiliations:** 1 Comparative Neuroanatomy Laboratory, and; 2 Zoocenoses Laboratory, Ecology Department, University of Calabria, Cosenza, Italy

**Keywords:** cadmium, diencephalon, endosulfan, histamine receptor subtype, mesencephalon, teleost fish

## Abstract

Common environmental contaminants such as heavy metals and pesticides pose serious risks to behavioral and neuroendocrine functions of many aquatic organisms. In the present study, we show that the heavy metal cadmium and the pesticide endosulfan produce such effects through an interaction of specific cerebral histamine receptor subtypes in the teleost ornate wrasse (*Thalassoma pavo*). Treatment of this teleost with toxic cadmium levels for 1 week was sufficient to induce abnormal swimming movements, whereas reduced feeding behaviors were provoked predominantly by elevated endosulfan concentrations. In the brain, these environmental contaminants caused neuronal degeneration in cerebral targets such as the mesencephalon and hypothalamus, damage that appeared to correlate with altered binding levels of the three major histamine receptors (subtypes 1, 2, and 3). Although cadmium accounted for reduced binding activity of all three subtypes in most brain regions, it was subtype 2 that seemed to be its main target, as shown by a very great (*p* < 0.001) down-regulation in mesencephalic areas such as the stratum griseum central layer. Conversely, endosulfan provided very great and great (*p* < 0.01) up-regulating effects of subtype 3 and 1 levels, respectively, in preoptic-hypothalamic areas such as the medial part of the lateral tuberal nucleus, and in the suprachiasmatic nucleus. These results suggest that the neurotoxicant-dependent abnormal motor and feeding behaviors may well be tightly linked to binding activities of distinct histamine subtypes in localized brain regions of the *Thalassoma pavo*.

A number of neurotoxic environmental contaminants, recognized as endocrine disruptors, have aroused much interest in the field of neuroendocrinology (Pillai et al. 2003). In particular, polycyclic aromatic hydrocarbons, polychlorinated biphenyls, and many heavy metals such as arsenic, cobalt, and mercury in the organic form appear to impair growth, development, and sociosexual behavior in vertebrates (Beauvais et al. 2001; Bisson and Hontela 2002). Cadmium is one of the heavy metals that pose an increasing health threat to ecologic communities and humans (Harvey et al. 1999). Because of widespread industrial applications such as the use of alloys for metal coatings and nickel-cadmium batteries as well as the burning of fossil fuels, urban traffic, and waste incineration, this pollutant is emitted into the atmosphere (Liao and Freedman 1998). It is taken up readily by humans and other mammals not only via inhalation but also via the food web (Waalkes et al. 1992). By binding to cysteine residues or generating reactive oxygen species (Risso-de Faverney et al. 2001), Cd has been shown to influence genomic and postgenomic processes in liver, kidney, lung, and brain (Minami et al. 2001). Similar alterations appear to be linked with neuronal dysfunctions in the hypothalamic–pituitary–testicular axis and inadequate neurosecretory activities of pituitary cells (Lafuente et al. 2001). Interestingly, these same degenerative processes in the preferential brain region, that is, the olfactory bulb, appear to corroborate evidence that foraging and aggression controlled by this region constitute vital behaviors in fish (Griffiths and Armstrong 2000; Scherer et al. 1997).

The persistence and accumulation of the insecticide endosulfan (6,7,8,9,10,10-hexachloro-1,5,5a,6,9,9a-hexahydro-6,9-methano-2,4,3-benzodioxathiepin-3-oxide) in human tissues are of concern (Martinez et al. 1997). Its resistance to biologic degradation and its low water solubility not only favor binding to soil particles and persistence within surface waters but also prompt bioconcentrations resulting in up to 600 times ambient water concentration in some species (Miller and Llados 1999). The purpose of this insecticide was to protect economically important crops such as tobacco and cotton; little attention was paid to neurologic risks to humans or other animals in terrestrial and aquatic ecosystems. Recent studies show that similar pesticides alter aggressive and reproductive behaviors in teleost fish (Carlson et al. 1998; Roex et al. 2001) through the interference of cerebral neuromediating systems (Clark 1997) such as the histaminergic system. This system is actively involved in blocking chemical-dependent stressful conditions such as writhing and consequently modifies responses to cold and immobilization states (Ferretti et al. 1998; Ito 2000).

The histamine (HA) receptor complex is one of the main neurosignaling systems that, in addition to “allergic” and anti-inflammatory processes, has been recognized for its role in many neurologic functions such as attention, arousal, cognition, movement, and feeding in mammalian species (Lin et al. 1996). Structurally, the histaminergic neuronal fibers, originating from the hypothalamus, are projected extensively throughout the central nervous system and promote their actions via three distinct receptor subtypes denoted H*_n_*R (H_1_R, H_2_R, H_3_R) (Hill et al. 1997). All but H_3_R subtypes are postsynaptically located and are coupled positively to adenylyl cyclase and phospholipase C, whereas H_3_R has been isolated at both the presynaptic and postsynaptic levels (Brown et al. 2001). Recently, Spieler et al. (1999) observed that inhibition of the H_1_R site is linked to the improvement of appetitive reversal learning and memory tasks in the goldfish *Carassius auratus*—a relationship that was further supported by the detection, via the application of their specific and selective antagonists, of H_3_R (Peitsaro et al. 2000) and H_1_R (Choich et al. 2004) in brain areas of the zebrafish *Danio rerio* and the tilapia *Oreochromis niloticus*, respectively. Moreover, identification of these subtypes in other vertebrates such as amphibians and reptiles (Brodin et al. 1990; Inagaki et al. 1991) is consistent with their highly conserved profile throughout vertebrate phylogeny (Kaslin and Panula 2001). On the basis of the above data, the intention of our study was to establish whether Cd and endosulfan neurotoxicologic effects on behavioral activities could be controlled through cerebral histaminergic neuronal mechanisms in the ornate wrasse. In fish, adaptations to environmental variables, including chemical–physical water properties such as temperature, photoperiod, ionic balance, and pollutants (Huang et al. 2004), are involved in the acceleration of the diandric-protogynic physiologic state, which makes fish valuable models for the investigation of neurologic adaptation mechanisms (Bass and Grober 2001; Larson et al. 2003). Furthermore, the host-cleaning symbiosis of the ornate wrasse *Thalassoma pavo* not only is critical for the stability of ecosystems (Zander and Sötje 2002) but also improves environmental conditions for commercially valuable fish and thus makes the ornate wrasse an important ecologic bio-marker species.

## Materials and Methods

### Animals.

We collected 32 young female ornate wrasses (body weight, 20–25 g; length, ~ 16–18 cm) from the Tyrrhenian Sea; they were acclimated to laboratory conditions for 1 week. During this period, fish were maintained in flow-through tanks containing 150 L of seawater (19–21°C, pH 7.8) under a 12/12-hr light/dark photoperiod and fed daily (10 g/kg body weight) with commercial food (Morubel, Milan, Italy) corresponding to 2% of biomass in the tank. Animal maintenance and experimental procedures were in accordance with the *Guide for Use and Care of Laboratory Animals* (European Communities Council Directive 1986), and efforts were made to minimize animal suffering and reduce number of specimens used.

### Experimental treatments and behavioral observations.

In the first part of the study, fish (*n* = 12) were exposed for 1 week to one of the two concentrations of Cd (CdCl_2_ · 2H_2_O; Sigma, Milan, Italy): a sublethal concentration (2.26 mg/L; *n* = 6) or a calculated maximum acceptable toxicant (MAT) concentration (11.32 mg/L; *n* = 6), which were both less than the 96-hr LC_50_ (lethal median concentration) value of 28.68 mg/L (Giusi et al. 2004) and the 96-hr LC_50_ value of 20.12 mg/L obtained in the white sea bass, *Lates calcarifer* (Thophon et al. 2003). This contaminant was prepared by dissolving CdCl_2_ · 2H_2_O in appropriate volumes of seawater. Other fish (*n* = 12) were exposed for the same length of time to two concentrations of endosulfan (Sigma): a sublethal concentration (0.2 μg/L; *n* = 6) and a calculated MAT concentration (1.3 μg/L; *n* = 6) that were both less than the 96-hr LC_50_ value of 3.30 μg/L (Giusi et al. 2004), and the 96-hr LC_50_ value varying between 1.4 and 1.5 μg/L for freshwater fish (Johnson and Finley 1980), sublethal and MAT concentrations that fall within the ranges reported in surface waters (0.039–0.205 μg/L) and after runoff water events (0.01–1.3 μg/L) from agricultural areas, respectively (Naqvi and Vaishnavi 1993). The pesticide was dissolved in seawater. We compared both treatment groups with controls (*n* = 8) consisting of fish maintained under identical conditions except that only vehicle was added to the tanks. During the entire experiment, accumulation of wastes and pathogens was avoided by replacing the tanks with fresh seawater every day. To reduce to a minimum the stressful conditions of this operation, we rapidly transferred the fish with a small fishing net to the tanks containing fresh seawater and either one of the two contaminants in an effort to achieve the intended nominal concentrations. Throughout the behavioral sessions, we checked the feeding habits of the ornate wrasse to ensure that the fish ingested water containing the neurotoxicant, although the uptake of these compounds primarily depends on their passage through the gill system (Thophon et al. 2003).

We assessed the behavior and mortality for all fish that received either Cd or endosulfan in four 1-hr sessions each day for 1 week. The motor and feeding behaviors that we analyzed included hyperactive movements consisting in either swimming toward the surface, swimming in the same direction, or “bumping” into each other or against the tank; assuming a “relaxed” position or simply being inactive; and hyper-ventilation, which is defined as the number of times that the operculum opens and closes in a 1-hr observation session. We also recorded feeding frequency and quantity (milligrams) of food ingested during each observation session. The quantity of food ingested was determined after the residual food recovered at the bottom of the tank was dried and weighed. The above motor activities and feeding behaviors of both treatment and control groups, estimated as mean activity per 24 hr ± SE, were recorded with a digital video camera (model TR 7000 E; Sony, Tokyo, Japan) and elaborated at a personal computer (Microsoft Windows XP; Microsoft Corp., Redmond, WA)) using EthoLog software (version 2.2.5; Visual Basic, São Paulo, Brazil) for behavioral analyses.

### Amino cupric silver staining.

To establish whether abnormal behavioral activities were related to neuronal damage, fish treated in the same manner as in the behavioral study with sublethal (*n* = 3) and MAT (*n* = 3) concentrations of Cd and endosulfan were decapitated and their brains quickly removed (within 30 sec) and stored at −40°C according to common cryostat procedures for unfixed brains (Canonaco et al. 1997). Brains were mounted on a freezing stage of a sliding cryostat (Microm-HM505E; Zeiss, Wallford, Germany), and a serial set of representative coronal sections (30 μm) was selected at an interval of 240 μm for amino cupric silver staining procedures according to previously published methods (de Olmos et al. 1994); we adapted the exposition time (25 min) to neutral red for the different brain sections of our fish species. This method, which has been used mainly for mammalian brain studies, proved to be appropriate for cerebral neuronal fields that have undergone degeneration processes. The brain sections were rinsed with distilled H_2_O, placed into dishes containing the preimpregnating solution (silver nitrate [AgNO_3_], distilled H_2_O, D,L-alanine, copper nitrate [Cu(NO_3_)_2_], cadmium nitrate [Cd(NO_3_)_2_], lanthanum nitrate [La(NO_3_)_2_], neutral red, pyridine triethanolamine, isopropanol), heated in a microwave oven (45–50°C) for 50 min, and cooled at room temperature for 3 hr. The sections were then rinsed in distilled H_2_O, and after a quick rinse in acetone they were placed in an impregnating solution AgNO_3_, distilled H_2_O, ethanol, acetone, lithium hydroxide (LiOH), ammonium hydroxide (NH_4_OH)] for 50 min, followed by a 25-min fixation in a reducer solution (formalin, citric acid monohydrate, ethanol, distilled H_2_O) at a temperature range of 32–35°C. These sections were left overnight in distilled H_2_O, and the next day they were placed in a first bleaching solution [potassium ferricyanide in potassium chlorate solution, lactic acid] for 60 sec at room temperature. Afterward, they were bleached in a second bleaching solution (potassium permanganate, sulfuric acid) for 60 sec and rinsed in distilled H_2_O. For the stabilization phase, sections were transferred in sodium thiosulfate solution and rinsed again in distilled H_2_O. Finally, they were immersed in a rapid fixer solution for 5 min and counterstained with 0.5% neutral red solution (Carlo Erba, Milan, Italy) for 25 min, dehydrated in ethanol (50–100%) and xylene, and mounted with DPX (*p*-xylene-bis[*N*-pyridinium bromide]; Sigma) for observations with a bright-field Dialux EB 20 microscope (Leitz, Stuttgart, Germany). The effects of both neurotoxicants on the argyrophilic reaction at the different brain levels were compared with controls that consisted of fish maintained under conditions identical to those of the two treatment groups except that only vehicle was added to the tanks. Because the same negative results were obtained at all brain levels, only two representative posterior areas were illustrated and compared with the different brain areas of the two treatment groups.

### Effects of Cd and endosulfan on H_1_R–H_3_R.

The neurotoxic actions of Cd and endosulfan were also correlated with the type of distribution pattern of the H*_n_*R neuronal system. Fish treated with the sublethal (*n* = 4) and MAT (*n* = 4) concentrations of Cd and endosulfan along with their control (*n* = 6) were used. The brains were removed and quickly frozen for storage at −40°C, and brain sections (14 μm thick) were thawed, dried at room temperature, and then handled according to *in vitro* binding studies for mammals (Ryu et al. 1996) that were adapted for fish brain sections (Peitsaro et al. 2000). Briefly, we incubated sections in 150 mM sodium potassium phosphate buffer (Sigma) 2 mM MgCl_2_ and 100 μM dithiothreitol pH 7.4 (Roche Diagnostic, Milan, Italy) containing different concentrations (0.5–20 nM) of [^3^H]-*N*-α-methyl-HA (NAMH; PerkinElmer Life Sciences, Boston, MA, USA). Some sections were incubated with 10 nM [^3^H]-NAMH using a wipe assay procedure. This concentration displayed the greatest affinity for H*_n_*R in the presence of the different values (1 μM–1 nM) of the following specific HA antagonists (Sigma): H_1_R antagonist pyrilamine, H_2_R antagonist cimetidine, and H_3_R antagonist thioperamide. Other sections were incubated with 10 nM [^3^H]-NAMH plus 500 μM of their corresponding antagonist for nonspecific binding values that proved to be similar to that of the background; subsequently an autoradiographic film (Hyperfilm; Amersham, Piscataway, NJ, USA) was apposed to dried sections and to slides containing plastic standards.

After an exposure period of 6 weeks (25°C), we developed the autoradiographic films according to previous methods (Canonaco et al. 1997) and we evaluated the different H_1_R–H_3_R binding densities, expressed in femtamole per milligram wet tissue weight, with a Panasonic Telecamera (objective lens FD; 50 mm, 1:3.5; Canon, Milan, Italy) attached to a Macintosh computer-assisted image analyzer system running Scion-Image 2.0 (National Institutes of Health Image, Bethesda, MD, USA). We stained labeled sections with cresyl violet acetate to identify the diencephalic, mesencephalic, and telencephalic brain regions, using the perch fish atlas (Cerdá-Reverter et al. 2001a, 2001b).

### Statistical analysis.

For the receptor binding study, Scatchard analyses of saturation binding data, which were fitted by a one-site and/or two-site model [based on the significance of extrasum squares using a LIGAND program (Munson and Rodbard 1980)] supplied relative affinity states and maximal receptor binding densities. To compare behavioral observations and histaminergic receptor binding data, we compared the treatment groups using a one-way analysis of variance (ANOVA) when there was a significant *p*-value ≤ 0.05, according to the Neuman-Keuls multiple-range post hoc test.

## Results

### Behavioral analysis.

**Figure 1:**
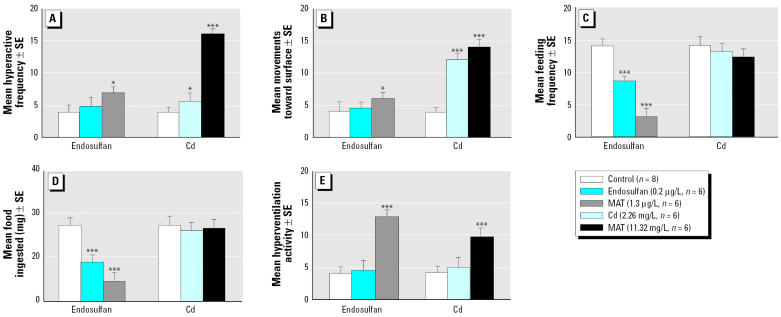
Assessment of effects of Cd and endosulfan on some motor activities of *Thalassoma pavo*: (*A*) hyperactive movements, (*B*) movements toward surface, (*C*) feeding frequency, (*D*) quantity (mg) of food ingested, and (*E* ) hyperventilation activity. For these behaviors a sublethal concentration of endosulfan and Cd as well as a MAT concentration of the two contaminants, respectively, were compared with controls. Values (means of activities/24 hr ± SE) of motor activities and feeding behaviors were estimated daily during four 1-hr observations for 1 week, as described in “Materials and Methods.” The behavioral data were analyzed by one-way ANOVA followed where necessary by post hoc Neuman-Keuls multiple-range test. *p < 0.05; ***p < 0.001.

Treatment of the ornate wrasse with Cd and endosulfan accounted for a net differentiation in the type of behavior responses. The MAT concentration of both stressors—11.32 mg/L and 1.3 μg/L, respectively—induced stereotype motor behaviors during the entire experimental session. Fish treated with Cd at 11.32 mg/L exhibited greater (*p* < 0.001; Figure 1A) hyperactive swimming activities such as moving in only a vertical direction and/or “bumping” against each other or against the glass tanks, in contrast to controls, which were often inactive and spent most of their time along the bottom of the tank. Fish treated with a concentration of 2.26 mg/L Cd displayed only moderate stereotype behaviors (Figure 1B), including hyperactive movements that consisted of swimming mainly in a vertical direction toward the surface of the water, whereas controls exhibited more random movements. Conversely, endosulfan caused a significant increase of some hyperactive movements (*p* < 0.05; Figure 1A,B) such as swimming in a vertical direction, whereas “bumping” type of swimming behaviors occurred in a less significant manner. This pesticide markedly reduced feeding, even at the lower concentration (0.2 μg/L). With both concentrations of endosulfan tested, feeding behavior was irregular, and overall, treated animals ate less food than did controls (Figure 1C,D). The MAT concentrations of both contaminants caused an excessive production of mucus on the operculum surface and, after 24 hr, hyperventilation became increasingly more severe up to the end of the study (Figure 1E).

### Analysis of amino cupric silver–stained tissue.

**Figure 2:**
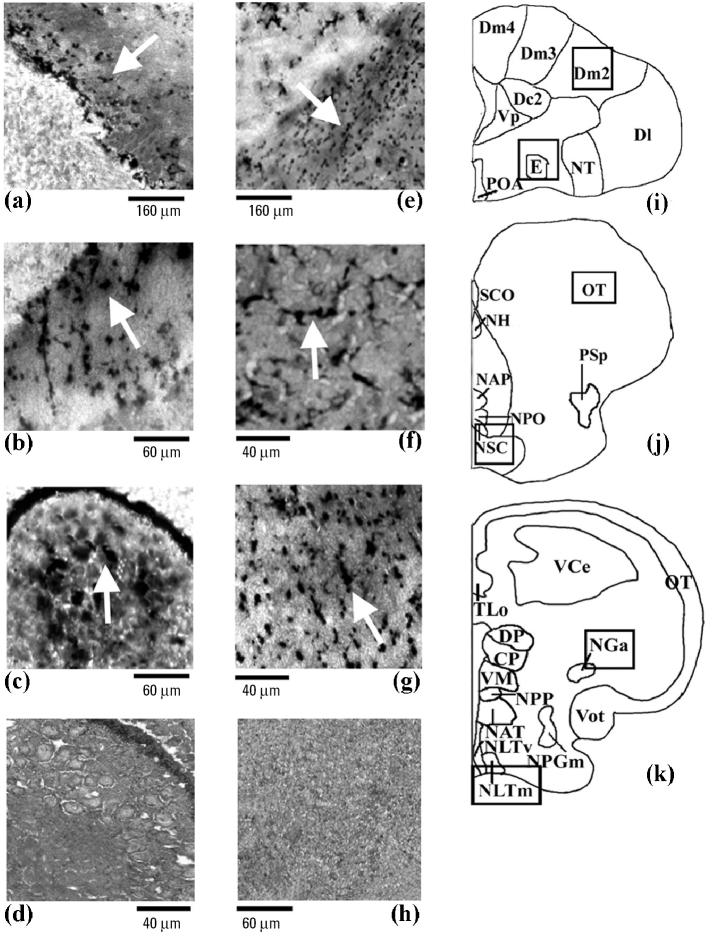
Photomicrographs showing the amino cupric silver staining pattern in rostral (*i*), middle (*j*), and posterior (*k*) brain areas of the *Thalassoma pavo*, treated with a MAT concentration of (*a–c*) Cd or (*e–g*) endosulfan. The effects of Cd (*n* = 6; arrows) were mostly observed in telencephalic and in mesencephalic areas such as Dm2 (*a*) and in the piriform SGC neurons of the optic tectum (*b*), respectively, and in the pretectal NGa (*c*), compared with control (*n* = 8); controls gave comparable results at all brain levels for both neurotoxicants as described in “Materials and Methods,” and so these same controls (*d*, *h*) were also used for the effects of endosulfan. In the case of endosulfan (*n* = 6), the major effects (arrows) were detected in the interneurons of the entopeduncular nucleus (*e*) and in the preoptic NSC area (*f*) and NLTm (*g*) of the hypothalamic lobe. Abbreviations: CP, central posterior thalamic nucleus; Dc2, central part of dorsal telencephalon, subdivision 2; Dl, lateral part of the dorsal telencephalon; Dm2–Dm4, medial part of the dorsal telencephalon, subdivisions 2–4; DP, dorsal posterior thalamic nucleus; E, entopeduncular nucleus; NAP, anterior periventricular nucleus; NAT, anterior tuberal nucleus; NGa, anterior part of the nucleus glomerulosus; NH, habenular nucleus; NLTm, medial part of lateral tuberal nucleus; NLTv, ventral part of lateral tuberal nucleus; NPGm, medial preglomerular nucleus; NPO, preoptic nucleus; NPP, posterior periventricular nucleus; NSC, suprachiasmatic nucleus; NT, nucleus taenia; OT, optic tectum; POA, preoptic area; PSp, parvocellular superficial pretectal nucleus; SCO, subcommissural organ; TLo, torus longitudinalis; VCe, cerebellum valvula; VM, ventromedial thalamic nucleus; Vot, ventral optic tract; Vp, postcommissural nucleus of the ventral telencephalon.

From the amino cupric silver staining analysis, it was possible to correlate these abnormal behaviors with evident neurodegeneration processes in telencephalic and mesencephalic regions. In particular, a MAT concentration of Cd supplied damaged external pyramidal neuron, as exhibited by a typically argyrophilic dark neuronal perikarya and often by a shrunken and folded appearance compared with little or no damage in controls (Figure 2d,h). This feature was limited mainly to the medial dorsal part of the telencephalon, subdivision 2 (Dm2; Figure 2a), and the pyramidal layer of the mesencephalic stratum griseum central (SGC; Figure 2b), which showed consistent dark axonal processes. The effects of Cd seemed to extend to other areas of the brain, namely, the anterior part of the nucleus glomerulosus (NGa; Figure 2c) of the diencephalic pretectal region that is involved, via mesencephalic circuits, with the regulation of visual motor functions in teleosts (Kaslin and Panula 2001). With endosulfan, substantial neurodegeneration was present in ventral telencephalic regions such as the entopeduncular nucleus (e; Figure 2e) plus the diencephalic suprachiasmatic nucleus (NSC; Figure 2f) and the medial part of lateral tuberal nucleus (NLTm; Figure 2g). In these brain regions endosulfan produced an altered pattern of neurons defined as an “interrupted string of pearls” as noted with degeneration of interneurons of mammals (Siegel et al. 1999).

### Effects of Cd and endosulfan on H_1_R–H_3_R.

**Figure 3:**
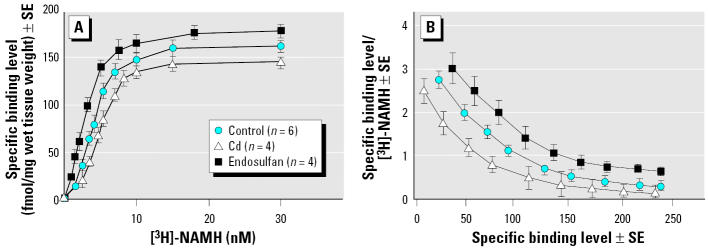
(*A*) A saturation curve of [^3^H]-NAMH binding (fmol/mg wet tissue weight ± SE), using wipe assays, was determined for the preoptic area of the *Thalassoma pavo* treated with MAT concentrations of Cd and endosulfan and compared with controls as described in “Materials and Methods.” (*B*) From the linear Scatchard plot, the negative slope was calculated to provide the mean dissociation constant (nM), whereas the intercept of the curve at the abscissa provided the maximal number of binding sites. Evaluation of saturation-binding study supplied similar results in three separate trials.

**Figure 4:**
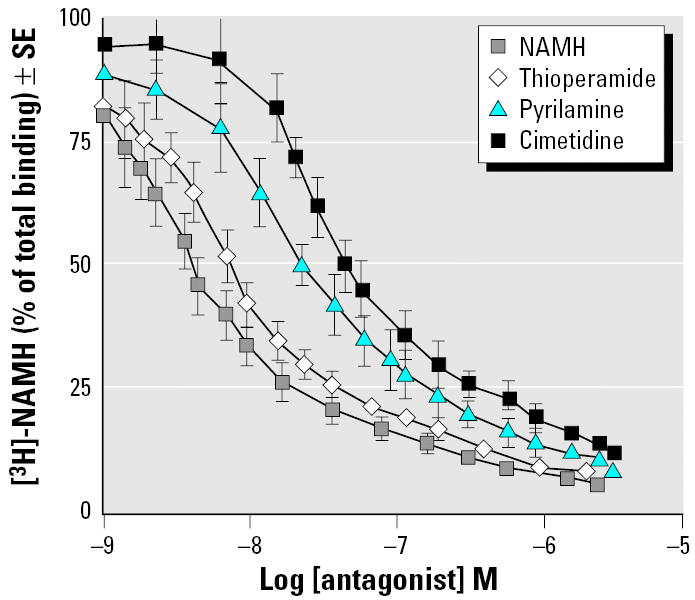
Displacement curves of [^3^H]-NAMH (% of total binding) in preoptic area of the *Thalassoma pavo* (*n* = 6) were generated in the presence of different concentrations (1 μM to 1 nm) of cold NAMH and of selective HA antagonists thioperamide, pyrilamine, and cimetidine as described in “Materials and Methods.” Each point represents mean ± SE of three separate tests.

**Figure 5:**
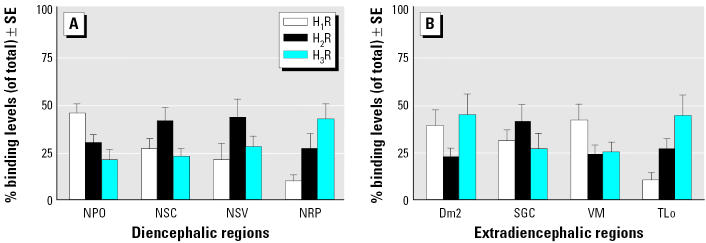
Percentage binding levels (of total) ± SE of H_1_R, H_2_R, and H_3_R sites in diencephalic (*A*) and extra-diencephalic (*B*) regions of the *Thalassoma pavo* (*n* = 6) were determined in the presence of their respective selective antagonists as described in “Materials and Methods.” Abbreviations: Dm2, medial part of the dorsal telencephalon, subdivision 2; NPO, preoptic nucleus; NRP, nucleus of the posterior hypothalamic recess; NSC, suprachiasmatic nucleus; NSV, nucleus of the saccus vasculosus; SGC, stratum griseum central; TLo, torus longitudinalis; VM, ventromedial thalamic nucleus.

When the regional distribution of HA receptors was determined in the presence of Cd and endosulfan, we observed a peculiar pattern of histaminergic expressing neurons in the same above brain regions of *Thalassoma pavo*. Such a relationship was based on a similar optimal [^3^H]-NAMH binding constant (Figure 3) in both treated and control fish with respect to that of rodents (unpublished data). Overall, the highest (> 140 < 200 fmol/mg wet tissue weight) HA binding densities were shown to be typical of rostral areas such as the preoptic nucleus (NPO) as well as the torus longitudinalis (TLo) and SGC of midbrain regions, whereas lower (> 70 < 110 fmol/mg wet tissue weight) binding densities were reported for the central nucleus of the ventral telencephalon and molecular stratum of the cerebellum. Application of the selective HA receptor antagonists enabled us to demonstrate that it was the diencephalic region that proved to be a preferential target of the major distribution differences of all subtypes (H_1_R–H_3_R), as displayed by notable displacement capacities of these subtypes in the preoptic area (Figure 4), as well as high H_1_R and H_2_R levels in areas such as NPO (45%) and in the nucleus of the saccus vasculosus (NSV; 43%), respectively (Figure 5). The subtype H_3_R was predominantly higher in some regions and especially in Dm2 (45%) of the telencephalon and in TLo (44%) of the mesencephalon.

**Figure 6:**
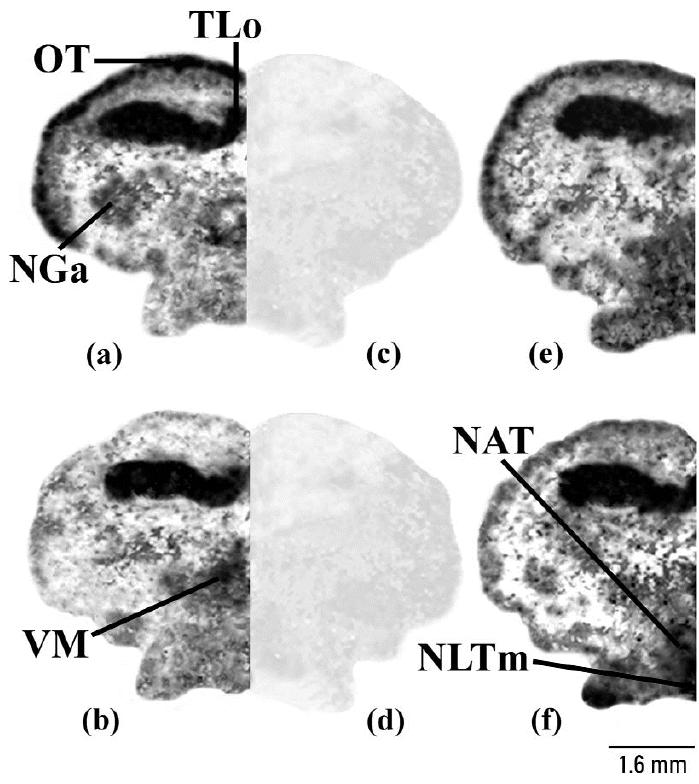
Representative binding autoradiograms displaying distinct receptor densities (black line) of H_2_R in the posterior regions of the *Thalassoma pavo* treated with a MAT concentration of Cd (*a*; *n* = 4) and of H_3_R in the same brain regions of animals that, instead, received a MAT concentration of endosulfan (*b*; *n* = 4), with respect to their corresponding (*e*, *f*) controls (*n* = 6). Binding pattern of these two subtypes appeared to be highly specific as shown by very similar background levels reported for [^3^H]-NAMH in presence of a 500× concentration of the selective antagonists cimetidine (*c*) and thioperamide (*d*), respectively, as described in “Materials and Methods.” Abbreviations: NAT, anterior tuberal nucleus; NGa, anterior part of the nucleus glomerulosus; NLTm, medial part of lateral tuberal nucleus; OT, optic tectum; TLo, torus longitudinalis; VM, ventromedial thalamic nucleus.

**Figure 7:**
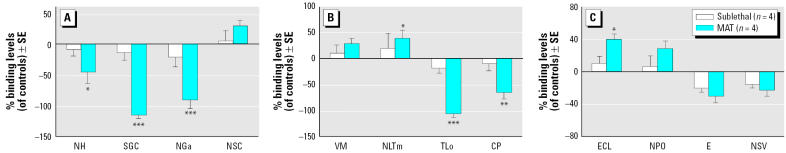
The effects of both sublethal and MAT concentrations of Cd on H_2_R (*A*), H_1_R (*B*), and H_3_R (*C*) with respect to their controls (*n* = 6) were expressed as a percentage binding level ± SE in the different brain regions of the *Thalassoma pavo*, as described in “Materials and Methods.” The levels were compared using one-way ANOVA followed where necessary by a post hoc Neuman-Keuls multiple-range test. *p < 0.05; **p < 0.01; ***p < 0.001. Abbreviations: CP, central posterior thalamic nucleus; E, entopeduncular nucleus; ECL, external cellular layer of olfactory bulb; NGa, anterior part of the nucleus glomerulosus; NH, habenular nucleus; NLTm, medial part of lateral tuberal nucleus; NPO, preoptic nucleus; NSC, suprachiasmatic nucleus; NSV, nucleus of the saccus vasculosus; SGC, stratum griseum central; TLo, torus longitudinalis; VM, ventromedial thalamic nucleus.

It is noteworthy that fish treated with a MAT Cd dose showed a down-regulating effect of H_2_R–expressing neurons, as displayed by the low binding densities in some midbrain regions of the representative autoradiograms (Figure 6). Of all the regions examined, SGC (−115%; *p* < 0.001) and NGa (−90%) of the mesencephalon (Figure 7A) seemed to contain the greatest down-regulating activities of H_2_R-producing neurons, whereas a moderate (*p* < 0.05) reduction was evident in the habenular nucleus (NH; −45%). A similar reduction that appeared to be also maintained for H_1_R-producing neurons and precisely a very great and great (*p* < 0.01) reduction of H_1_R levels in TLo (−105%) and the central posterior thalamic nucleus (CP; −65%), respectively, whereas a moderate up-regulating activity was instead detected for NLTm (+38%; Figure 7B). The subtype H_3_R did not appear to be a major target of Cd (Figure 7C) aside from the moderately higher levels (+40%) obtained in the external cellular layer (ECL) of the olfactory bulb.

**Figure 8:**
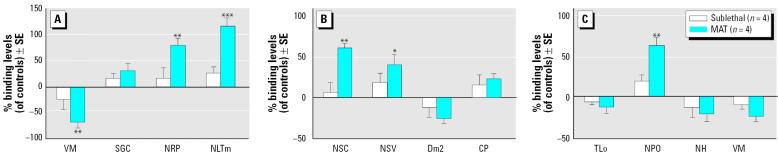
The effects of both sublethal and MAT concentrations of endosulfan on H_3_R (*A*), H_1_R (*B*), and H_2_R (*C* ) with respect to their controls (*n* = 6) were expressed as a percentage binding level ± SE in the different brain regions of the *Thalassoma pavo*, as described in “Materials and Methods.” The levels were compared using one-way ANOVA followed where necessary by a post hoc Neuman-Keuls multiple-range test. *p < 0.05; **p < 0.01; ***p < 0.001. Abbreviations: CP, central posterior thalamic nucleus; Dm2, medial part of the dorsal telencephalon, subdivision 2; NH, habenular nucleus; NLTm, medial part of lateral tuberal nucleus; NPO, preoptic nucleus; NRP, nucleus of the posterior hypothalamic recess; NSC, suprachiasmatic nucleus; NSV, nucleus of the saccus vasculosus; SGC, stratum griseum central; TLo, torus longitudinalis; VM, ventromedial thalamic nucleus.

The effects of endosulfan appeared instead to be preferentially directed toward H_3_R-producing neurons, as shown by the greater binding densities in the representative autoradiograms of midbrain regions (Figure 6b). The diencephalic region (Figure 8A) provided very great up-regulating effects, especially in the NLTm (+110%) and the nucleus of the posterior hypothalamic recess (NRP; +78%). By contrast, greatly decreased levels were detected in another hypothalamic area, that is, the ventromedial thalamic nucleus (VM; −70%). Moreover, the H_1_R-producing neurons of this brain region were a preferred target for endosulfan effects (Figure 8B), as indicated by the greatly increased levels in the NSC (+68%) plus moderately higher levels in the NSV (+40%). Conversely, the other subtype (H_2_R) did not prove to be a preferred target of this pesticide (Figure 8C) despite the moderately higher H_2_R levels in NPO (+60%).

## Discussion

We describe here for the first time neurotoxicologic effects of the heavy metal Cd and the insecticide endosulfan that are responsible for abnormal motor and feeding behaviors through histaminergic mechanisms in the ornate wrasse. A first abnormal behavior consisted of stereotype motor activities such as swimming in a constant direction or “bumping” against each other and/or against the glass tank, especially when the fish were treated with a MAT Cd concentration. These abnormal behaviors, as reported previously for *Thalassoma pavo* observed under field conditions (Giusi et al. 2004) and in a wide variety of fauna ranging from terrestrial vertebrates such as rodents (Lafuente et al. 2001) to aquatic species such as amphibians (James et al. 2004) and Chordata Ascidaecea (Bellas et al. 2001), should not be surprising because of wide distribution of this heavy metal in the different ecosystems. This condition appears mainly in aquatic communities because Cd readily accumulates in the different tissues after uptake via the calcium transport pathway of gill’s chloride cells (Wood 2001), above all in the olfactory structures that are considered to be its preferential target (Tallkvist et al. 2002). In this context the interference of such sensory communicating structures in the ornate wrasse may offset normal responses to olfaction-mediated stimuli such as migration and physical contact with other fish, which is in accordance with the irregular responses to alarm signals as well as modification of aggressive social relationships that have been reported in rainbow trout treated with toxic Cd doses (Scott et al. 2003).

In pesticides both sublethal and MAT concentrations of endosulfan caused abnormal feeding behaviors, whereas altered swimming movements were less evident than in Cd-treated animals. As a consequence, the consumption of food at an asynchronous rhythm and at different time intervals is in good agreement with other pesticides, accounting for feeding difficulties via neuronal functional hindrances in the goldfish (Bretaud et al. 2000). Similar difficulties obtained even under sublethal concentrations tends to suggest that sensorimotor threshold activities are susceptible to this contaminant, as shown by “startled” motor behaviors being tightly associated with the olfactory-dependent neuromediation of optomotor responses such as predation, foraging, and orientation toward food odor (Pan and Dutta 1998). These olfactory-dependent responses appear to be determining elements for feeding behaviors throughout the various biologic phases of the fish, as demonstrated by both young and adult Japanese killifish being neither attracted to nor able to consume food after receiving similar endosulfan doses (Gormley and Teather 2003).

When the toxicologic actions of both environmental contaminants were assessed at the structural level of the brain, notable neuro-degenerative events were observed, as shown by the diffused amino cupric silver staining of neurons in the different brain regions. With this method it was possible not only to immediately and clearly detect the precise location of neuronal trauma (Siegel et al. 1999) but also to distinguish between somata and axonal damage in some diencephalic, mesencephalic, and telencephalic sites of *Thalassoma pavo*. Of the brain regions exposed to MAT Cd concentrations, the telencephalic Dm2 displayed the greatest axonal fiber damage and interstitial edema. This condition fits nicely with the infiltration properties of the heavy metal in mammalian telencephalic regions such as the hippocampus (Mendez-Armenta et al. 2001), which is involved in analogous functions such as learning, spatial memory, and motor behaviors that are controlled by Dm2 in fish (Portavella et al. 2004). Even SGC and NGa of mesencephalic and pretectal areas, respectively, which are related to the modulation of multisensorial inputs (visual, acoustic, and electroreceptive signals), supplied perturbed dendritic spine formation and deformed soma in a fashion similar to that of mesencephalic trigeminal neurons of rodents exposed to Cd (Yoshida 2001). The effects of endosulfan were instead involved mainly with axonal deformations of interneurons in diencephalic areas such as NSC and preoptic area of the hypothalamus, an effect that tends to overlap cellular alterations and interstitial infiltration events induced by the pesticide carbofuran in teleosts (Ram et al. 2001). Ram et al. (2001) also showed that such a contaminant was responsible for a reduction in number and size of neurons and consequently altered neurotransmission functions in this same brain region.

Interestingly, the neuronal alterations provoked by both environmental contaminants in the present study seemed to coincide with changes of the histaminergic transcriptional activities in some telencephalic and mesencephalic regions and in the anterior and posterior areas of the hypothalamus. The hypothalamic area is considered to be a key production site of HA not only in mammals (Pillot et al. 2002) but also in amphibians (Airaksinen and Panula 1990) and in some fish species such as the zebrafish (Kaslin and Panula 2001). Because toxicologic effects of Cd and endosulfan occur in distinct and localized brain regions seems to support strongly a behavior-linked relationship of these neurotoxins, as demonstrated by Cd being preferentially directed toward the motor-controlling cerebral regions and endosulfan being involved predominantly on endocrine-dependent activities of hypothalamic areas. The effects of Cd exposure on pretectal and tegmental areas are characterized primarily by a down-regulatory activity of H_2_R-expressing neurons, whereas a similar activity of H_1_R-expressing neurons was detected for TLo and CP. Moreover, on the basis of the low levels of H_2_R occurring not only in key motor telencephalic areas but also in mesencephalic and cerebellar regions of the mormyrid electric fish (Han et al. 2000) and of other vertebrates (Minami et al. 2001), it appears that a down-regulation of this subtype might represent an important condition of the histaminergic inhibitory effects on locomotor behaviors (Santos et al. 2003). The inhibitory effects may be accomplished by the regulation of parameters such as swimming velocity, location of objects, and overall vestibular activities that are controlled by these same brain regions (Meek 1990; Xue et al. 2003).

Conversely, endosulfan appeared predominantly to promote enhanced levels of H_3_R-expressing neurons mainly in hypothalamic areas such as NLTm and NRP as well as of H_1_R-expressing neurons in NSC. This relationship appears to be strengthened by decreased swimming and feeding behaviors obtained immediately (after 2 hr) in the Chinook salmon when treated with the organophosphate pesticide diazinon (Scholz et al. 2000). The finding that the diencephalic region is a major target of pesticide toxic effects should not be surprising because polychlorinated biphenyls interfere with other hypothalamic activities, including the regulation of body temperature and the activities of the hypothalamic–pituitary–gonadal circuits, with severe consequences on reproductive and hormone-releasing activities (Bloomquist 2003; Cooper et al. 2000). It is noteworthy that high levels of H_3_R-expressing neurons have been correlated with a reduction of food intake through the suppression of appetite and energy expenditure in the same hypothalamic areas (Takahashi et al. 2002). In addition, the high levels of H_1_R-expressing neurons in other hypothalamic sites of the ornate wrasse plus the inhibition of these subtypes accounting for improved feeding habits in the goldfish (Spieler et al. 1999) appear to be consistent with an important inhibitory role of H_1_R and H_3_R, at least in hypothalamic nuclei of this teleost.

In conclusion, these results provide direct evidence that the toxicologic risks of endosulfan and Cd on the motor and feeding behavior of *Thalassoma pavo*, as shown by evident morphologic neuronal damages and distinct H*_n_*R-expressing patterns, appear to be very strongly correlated with histaminergic neurosignaling mechanisms. Although most research to date has mostly considered the physiologic risks of the environmental toxicants, here we show that the abnormal behaviors could be linked to specific HA subtype interactions operating in some cerebral regions, at least in the ornate wrasse. Consequently, the motor activities appear to be tightly linked to Cd via variations of mainly H_2_R-expressing neurons in the mesencephalic and telencephalic regions, whereas modified feeding behaviors induced by endosulfan seem to be related to the differences of H_1_R- and H_3_R-expressing neurons mainly in hypothalamic areas. We are still at the beginning of this research, but molecular neuronal interests directed toward the role of environmental disruptors on aquatic organisms could provide further insights regarding not only the behavioral hazards of these contaminants but also neurotoxic mechanisms operating during the entire development cycle of fish, with the intent of minimizing ecologic and commercial risks of this very important class of vertebrates.
